# Determinants of health-related quality of life in patients with celiac disease: a structural equation modeling

**DOI:** 10.1186/s12955-021-01842-5

**Published:** 2021-08-24

**Authors:** Zeinab Nikniaz, Mohammad Asghari Jafarabadi, Mahdieh Abbasalizad Farhangi, Masood Shirmohammadi, Leila Nikniaz

**Affiliations:** 1 Liver and Gastrointestinal Diseases Research Center, Tabriz University of Medical Sciences, Tabriz, Iran; 2 Road Traffic Injury Research Center, Tabriz University of Medical Sciences, Tabriz, Iran; 3 Community Nutrition Department, Tabriz University of Medical Sciences, Tabriz, Iran; 4 Tabriz Health Services Management Research Center, Tabriz University of Medical Sciences, Tabriz, Iran

**Keywords:** Coeliac disease, Quality of life, Structural equation modeling

## Abstract

**Background:**

We aimed to investigate the determinants of Health-related quality of life (HRQOL) in Iranian patients with celiac disease (CD) using the structural equation modeling (SEM).

**Methods:**

In the present cross-sectional study, a total of 170 adult patients with CD were recruited. The information regarding adherence to diet, symptom severity, and HRQOL were collected using the celiac disease adherence test (CDAT), gastrointestinal symptom rating scale (GSRS), and SF-36 questionnaire respectively. Association between various studied variables and HRQOL was assessed using SEM. The standardized regression weights were used to assess total, direct and indirect effects. The model fit indices were used to assess the “goodness of fit” between the hypothesized models.

**Results:**

The mean age of participants was 37.57 ± 9.59 years. The results of SEM indicated that the overall fit of our model was acceptable. Adherence to the diet, GSRS score, occupation, and education level was significantly related to PCS of SF-36; and adherence to the diet, GSRS score, and education level were significantly correlated with MCS of SF-36. The analysis of indirect associations indicated that only adherence to diet indirectly via GSRS score was significantly associated with PCS and MCS of SF-36.

**Conclusion:**

In adult patients with celiac disease, HRQOL was associated with age, education, adherence to GFD, and GSRS score. Additionally, occupation and disease duration were associated with HRQOL only in women and men respectively.

## Background

Celiac disease (CD) is a common disorder that is caused by autoimmunity to the gluten protein. It affects about 0.7 to 1% of the population worldwide [1] and its prevalence in Iran is about 2% [2]. Eliminating Gluten-containing foods and products is the only effective and safe treatment [3]. Considering the chronic nature of this disease and since lifelong adherence to gluten-free diet (GFD) is demanding and costly, the quality of life of these patients is affected.

Different demographic, disease related and treatment–related factors can affect the quality of life in patients with celiac disease. One of the important factors is adherence to GFD. In various studies in children and adults, it has been shown that adherence to a gluten-free diet (GFD) had a positive effect on improving symptoms and health-related quality of life (HRQOL) in patients with celiac disease [4–6]. However, considering the limitations of this diet, it can also negatively affect the quality of life. In addition to the treatment effect, other determinant factors were also reported that affect the quality of life in these patients. These factors including demographic factors such as education level, employment status; and disease-related factors such as the presence of comorbidities and disease duration, and presence of symptoms [7, 8]. For example, in a study in Spain, it has been shown that age, gender, and GFD duration were factors that independently associated with HRQOL in Spain [9]. In addition, previous studies have indicated that symptom severity also affects the quality of life in celiac patients. For instance, Usai et al. indicated that there was a negative association between the number of symptoms and comorbidities and HRQOL in patients with celiac disease [10].

To the best of our knowledge, no study in Iran has assessed the determinant factors that affect the HRQOL in Iranian celiac patients. Moreover, most of the previous studies in patients with celiac disease have only reported the direct correlation between different factors and HRQOL. By means of this method, only the direct association of various variables on the outcome can be assessed. However, the exploration of indirect effects could offer new findings in the relationship between different demographic and disease-related factors and HRQOL in patients with celiac disease. So, in the present study, we used structural equation modeling that enables analysis of the interrelationship of independent variables and their direct and indirect associations through other variables. Considering that the HRQOL is affected by different variables, defining its determinants through this technique is beneficial.

Therefore, for the first time, we employed the structural equation modeling (SEM) technique for investigating the determinants of HRQOL in Iranian patients with celiac disease. In the current investigation, we analyzed the relationship between different demographic and disease-related factors and HRQOL in patients with celiac disease; and applied the SEM technique to determine the effect of these variables on HRQOL in a hypostatized model.

## Materials and methods

In the present cross-sectional study, the adult patients were selected randomly from the East-Azerbaijan celiac disease registry database. In this registry, the patients were registered if they have a positive serology test confirmed by compatible duodenal histological findings. The patients were included in the present study if they aged more than 18 years old, and were on GFD for at least 6 months.

The sample size for the present study was calculated based on the Bentler recommendation [11] with a minimum of ten observations per estimated parameters. So, we needed a sample size of 90 participants at least. In the present study, a total of 170 participants was recruited.

The Ethics Committee of Tabriz University of medical sciences (IR.TBZMED.REC.1399.904) approved the study and all participants gave written informed consent.

### Variables

The information regarding demographic characteristics including age, education level, employment status, marital status was obtained by an expert researcher using a questionnaire.

Medical records were used to gather information regarding comorbidities such as diabetes mellitus, cancers, chronic kidney diseases, chronic liver diseases, autoimmune diseases, inflammatory diseases, and psychological disorders.

Adherence to diet was assessed using the celiac disease adherence test (CDAT) questionnaire [12]. This questionnaire assesses the level of adherence to gluten-free diet using seven questions on the five Likert scale and the total score ranged between seven and 35. We considered a CDAT score of < 13 as good adherence, 13–17 as moderate adherence, and > 17 as poor adherence to GFD [13]. This questionnaire was previously translated to Persian, and its validity was confirmed in the previous study [12].

The severity of gastrointestinal symptoms was assessed by the Persian version of the gastrointestinal symptom rating scale (GSRS) questionnaire [14]. This questionnaire includes fifteen questions on a seven-point Likert scale and higher scores indicate more severe symptoms. The questionnaire assesses five domains including diarrhea, constipation, abdominal pain, reflux, and indigestion.

The HRQOL was assessed using the SF-36 questionnaire. This questionnaire is a 36-item questionnaire that assesses physical [Physical Component Summary (PCS)) and psychological (Mental Component Summary (MCS)] health with a higher score indicating better health. This questionnaire was previously translated to Persian, and its validity was confirmed [15].

### Statistical analysis

**Fig. 1:**
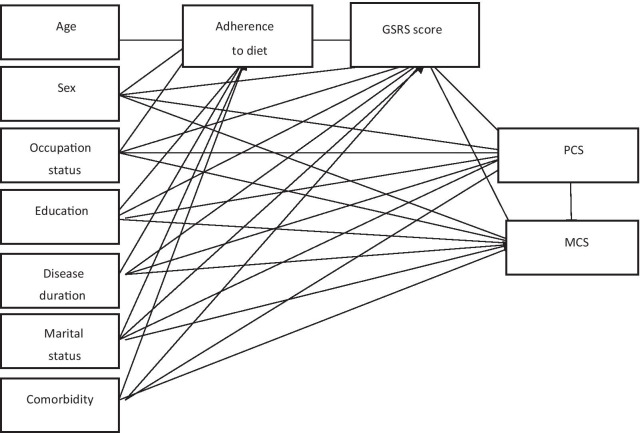
Hypothesized structural models

All analysis was performed using STATA-16. The normality of data distribution was assessed using the Kolmogorov–Smirnov test. The continuous variables were presented as mean ± SD and the categorical variable was presented as frequency (%). An independent t-test and chi-square were used to compare the continuous variables and nominal and categorical variables between males and females respectively. Association between sociodemographic factors, disease-related factors, and quality of life was assessed using structural equation modeling (SEM). As can be seen in the conceptual model (Fig. 1), adherence to diet and GSRS score were considered mediators. The standardized regression weights were used to assess the total, direct and indirect effects of variables on the HRQOL. The model fit was assessed to determine the “goodness of fit” between the hypothesized model and the data by use of several methods including the ratio of chi-square to the degree of freedom, root-mean-squared error of approximation (RMSEA), comparative fit index (CFI); and standardized root mean squared residual (SRMR). The acceptable values were Chi-square/DF < 5, RMSEA < 0.08, CFI > 0.9, SRMR < 0.08. A p value less than 0.05 was considered significant.

## Results

In the present study, 170 patients with celiac disease with a mean age of 37.57 ± 9.59 were studied. Of them, 103 (60.58%) were female, and 119 (70%) were married. The mean disease duration was 4.52 ± 3.36 years. There were statistically significant differences between males and females regarding marital status, employment status, mean PCS and MCS, and CDAT scores (Table 1).

**Table 1:** Baseline demographic and clinical characteristics of patients with celiac disease

Variable	Total (n = 170)	Male (n = 67)	Female (n = 103)	p value
Age (years)	37.57 ± 9.59	36.12 ± 10.44	38.9 ± 9.04	0.19
Education status n (%)				
≤ Diploma	97 (57.05)	33 (49.2)	64 (62.1)	0.07
College	73 (42.9)	34 (50.7)	39 (37.8)	
Marital status n (%)				
Single	51 (30.0)	28 (41.7)	23 (22.3)	0.004
Married	119 (70.0)	39 (58.2)	80 (77.6)	
Employed	83 (48.8)	50 (74.6)	33 (32.03)	< 0.001
Disease duration (year)	4.52 ± 3.36	4.28 ± 3.66	4.65 ± 3.19	0.54
Family history of CD, n (%)	13 (7.6)	6 (8.9)	7 (6.7)	0.79
CDAT score	15.55 ± 4.04	14.29 ± 3.59	16.26 ± 4.13	0.005
Adherence to diet	40 (23.5)	22 (32.8)	18 (17.4)	0.10
Presence of comorbidities, n (%)	51 (30)	21 (31.9)	30 (29.1)	0.41
GSRS score	30.18 ± 20.54	27.70 ± 19.96	31.59 ± 20.85	0.30
PCS	235.26 ± 92.88	266.27 ± 87.54	217.71 ± 91.70	0.004
MCS	218.01 ± 87.84	240.81 ± 90.57	205.10 ± 84.09	0.02

GSRS, Gastrointestinal Symptom Rating Scale; PCS, physical component summary; MCS, mental component summary; CDAT, celiac disease adherence test

### Structural equation modeling

In the present study, the overall fit of our model was acceptable (RMSEA [95% CI] 0.00 [0.00, 0.10]; Chi-square:2.24; DF: 4, Chi-square/df: 0.56; CF:1; and SRMR:0.02).

**Fig. 2:**
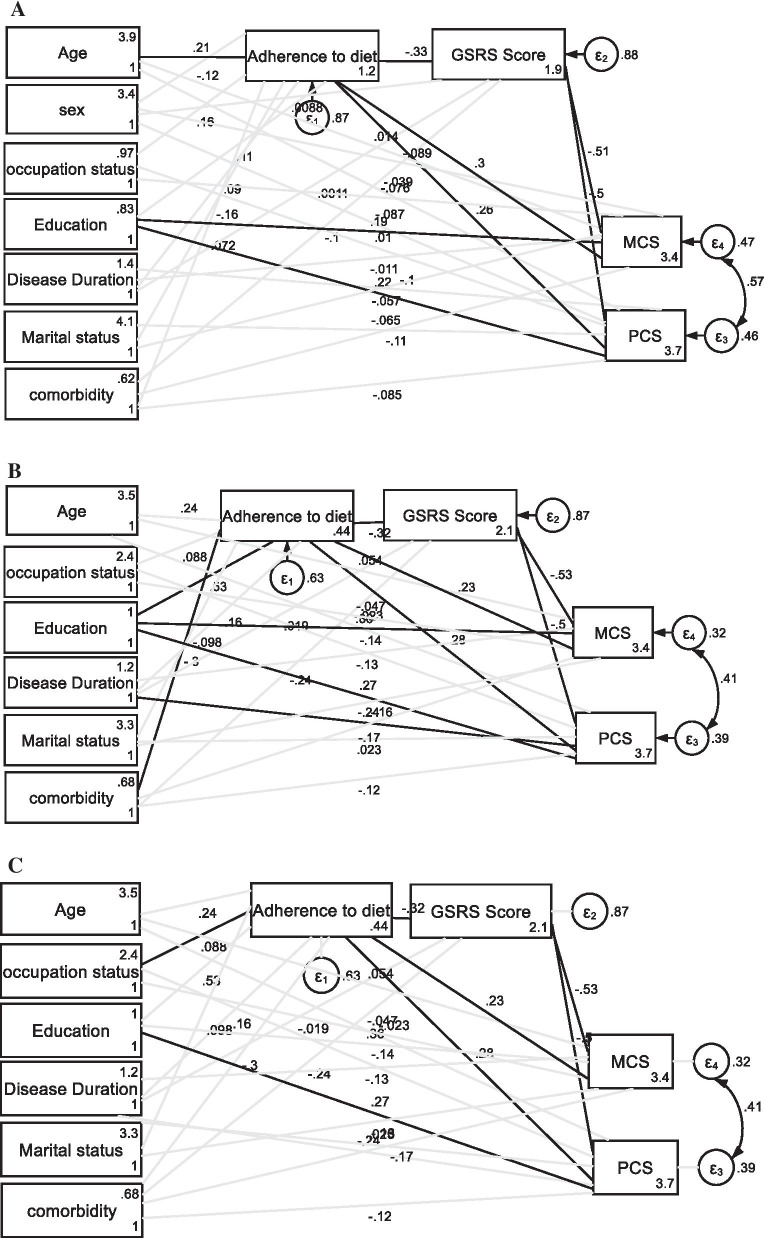
Final structural models after testing the association between socio-demographic and clinical factors and quality of life in total population (**a**), males (**b**), and females (**c**). Black lines indicate significant associations and gray lines indicate insignificant association

As presented in Table 2, the result of SEM analysis indicated that adherence to the diet, GSRS score, occupation, and education level were significantly related to PCS of SF-36; and adherence to the diet, GSRS score, and education level were significantly correlated to MCS of SF-36. The analysis of indirect association indicated that only adherence to diet indirectly via GSRS score was significantly associated with PCS and MCS of SF-36 (Fig. 2a).

**Table 2:** Total, direct and indirect effects of independent variables on dependent variables

Variables	SF-36 domains	Effect	Age SPC	Sex	Occupation	Education level	Marital status	Disease duration	Comorbidity	Adherence to diet	GSRS
			SPC	SPC	SPC	SPC	SPC	SPC	SPC	SPC	SPC
Total	PCS	Total	0.47	− 26.94	14.42*	50.62*	− 28.76	− 1.73	− 13.37	52.43*	− 2.27*
		Direct	− 0.37	− 16.78	1.85	41.86*	− 13.98	− 2.75	− 17.58	31.85*	− 2.27*
		Indirect	0.85	− 10.15	12.57	8.76	− 14.78	1.02	4.21	20.57*	
	MCS	Total	1.02	− 26.67	− 0.17	42.41*	− 27.15	0.80	− 18.65	54.95*	− 2.18*
		Direct	0.13	− 16.10	− 13.35	33.23	− 11.65	− 0.27	− 22.17	35.15*	− 2.18*
		Indirect	0.89	− 10.57	13.17	9.18	− 15.49	1.07	3.51	19.79*	
Males	PCS	Total	0.67		− 24.34	87.45	− 3.60	− 3.82	− 25.29	55.49	− 2.20
		Direct	− 0.19		− 33.76	46.87	4.02	− 5.67	− 22.96	35.18	− 2.20
		Indirect	0.87		9.41	40.58	− 7.62	1.84	− 2.33	20.30	
	MCS	Total	1.27		− 3.08	103.104	− 35.78	− 1.47	− 31.34	51.69	− 2.39
		Direct	0.46		− 11.86	12.03	− 28.68	− 3.22	− 32.80	29.61	− 2.39
		Indirect	0.81		8.77	37.80	− 7.10	1.75	1.25	22.07	
Females	PCS	Total	− 0.06		43.95	18.72	− 39.38	− 0.44	2.91	55.49	− 2.45
		Direct	− 0.87		18.25	33.84	− 23.50	0.29	− 1.64	25.31	− 2.45
		Indirect	0.80		25.70	− 15.11	− 15.88	− 0.73	4.56	23.25	
	MCS	Total	0.66		30.04	− 3.27	− 16.72	0.93	− 1.78	49.16	− 2.14
		Direct	− 0.15		4.01	12.03	− 0.64	1.56	− 6.21	28.78	− 2.14
		Indirect	0.81		26.02	− 15.30	− 16.10	− 0.62	4.43	20.38	

GSRS, Gastrointestinal Symptom Rating Scale; PCS, physical component summary; MCS, mental component summary; CDAT, celiac disease adherence test; SPC, standardized path coefficient

^*^Indicates significant correlation

We also analyzed the association between independent variables and PCS and MCS in males and females separately (Fig. 2b, c). As can be seen, in males, adherence to the diet, GSRS score, disease duration, and education level were directly related to PCS of SF-36; and adherence to diet and GSRS score were directly related to MCS of SF-36. The analysis of the indirect association indicated that education level and adherence to diet were also indirectly related to the PCS and MCS of SF-36.

In females, CDAT, GSRS, and education level were directly associated with PCS of SF-36; and adherence to diet and GSRS score were significantly associated with MCS of SF-36. The analysis of the indirect effect revealed that occupation and adherence to diet were significantly associated with PCS and MCS score of SF-36 (Fig. 2c).

## Discussion

In the present study the result indicated that in the whole population, adherence to the diet, GSRS score, occupation, and education level was directly associated with PCS and MCS of SF-36. Previously, a meta-analysis study on 436 patients with celiac disease, showed that dietary adherence to GFD was significantly associated with both SF-36 MCS and SF-36 PCS [4]. The direct association of dietary adherence with quality of life may partly be due to reductions in depression. Previous studies indicated that GFD had a significant positive effect on depression in patients with celiac disease [16, 17]. In the present study, we also showed that in addition to the direct association, adherence to the diet was indirectly associated with PCS and MCS of SF-36 via GSRS score. Previously, it has been shown that complete adherence to the GFD may be associated with mucosal healing, which resulted in improvement of symptoms [18].

In the present study, we also showed that education level had a direct association with both components of quality of life. Moreover, in males, it was indirectly associated with the quality of life. Previous studies also showed a significant association between education level and HRQOL [19]. It has been indicated that education allows for the development of cognitive skills related to personal care [20]. Moreover, a low level of education intensifies the complications of chronic diseases because of a lack of knowledge [21]. It has been indicated that the education level is related to the higher socioeconomic status [22]. In a previous study, it has been shown that income level has a significant association with health-seeking behavior and access to health care [23], which are correlated with a higher quality of life. In addition to the direct association, education level was indirectly associated with quality of life via an effect on adherence to the diet. Previous studies in celiac disease and other diseases indicated that a high level of education has a positive association with adherence to dietary recommendations [24]. A higher education level was correlated with better adherence to the diet via higher household income and also better self‐perceived knowledge of the GFD.

We found that in males, disease duration had a negative direct association with PCS of SF-36. This finding was similar to the result of the study in Spain [9]. In other chronic diseases such as diabetes similar finding was reported [25]. In the present study, 84.6% of patients had a disease duration of more than one year. We postulated that since strict compliance to GFD may be difficult and costly, the disease may cause significant limitations and impairment of patients’ lifestyles in the long term.

In females, occupation status had a direct positive association with both domains of quality of life. Previous studies in the women population also showed a similar result in different health conditions [26–30]. In women who are working, the social well-being and functional well-being may be higher. This may be related to the greater social support available from coworkers and friends in the workplace and having a sense of normalcy due to their ability to work [31]. Besides, GFD treatment is costly [32–34]. So, employment could have a direct economic effect on preparing gluten-free foods.

This study had the following limitations. The data for this study was obtained cross-sectionally, and the causality could not be inferred. The data about the quality of life was obtained using a general questionnaire, not diseases specific questionnaire. However, this questionnaire was valid and used in many previous studies to assess the quality of life in celiac patients [4, 6, 35]. We just assessed the association between demographic and disease characteristics and quality of life in the East Azerbaijan population. Moreover, the included population is rather young. These issues may normally restrict the generalization of the results. However, the mean age was almost similar to the mean age (35.08 ± 16.78 years) of all patients who were registered in the East Azerbaijan celiac disease registry system. In addition, in the subgroup analysis, the number of male participants was rather low that may limit the power of analysis in this subgroup. However, some studies recommended at least five participants per variable [36]. Based on this recommendation, the number of males was not very low in the present study.

## Conclusion

The result of SEM indicated that in adult patients with celiac disease, HRQOL was affected by age, education level, adherence to GFD, and GSRS score. Additionally, occupation and disease duration were associated with HRQOL only in women and men respectively. This structural model provides beneficial information for planning future health promotion programs in celiac patients. Moreover, considering the nature of determinants of QoL in celiac patients, the level of adherence to GFD can be studied in future interventional studies.

## Data Availability

The datasets generated and/or analyzed during the current study are not publicly available due to the institution's policy, but are available from the corresponding author upon reasonable request.
